# Spatiotemporal Distribution of Human Rabies and Identification of Predominant Risk Factors in China from 2004 to 2020

**DOI:** 10.1371/journal.pntd.0012557

**Published:** 2024-10-31

**Authors:** Weiwei Meng, Tianren Shen, Okugbe Ebiotubo Ohore, Susan Christina Welburn, Guojing Yang

**Affiliations:** 1 NHC Key Laboratory of Tropical Disease Control, School of Tropical Medicine, the First Affiliated Hospital, Hainan Medical University, Haikou, Hainan, China; 2 School of Public Health, Hainan Medical University, Haikou, Hainan, China; 3 Zhejiang University-University of Edinburgh Institute (ZJU-UoE Institute), Zhejiang University, International Campus, Haining, China; 4 Infection Medicine, Deanery of Biomedical Sciences, Edinburgh Medical School, College of Medicine and Veterinary Medicine, The University of Edinburgh, 1 George Square, Edinburgh, Scotland, United Kingdom; Universidad Nacional Mayor de San Marcos, PERU

## Abstract

Human rabies is a prevalent issue in China, posing a significant public health concern in the country. This study fitted the Bayesian model of separable in spatial and temporal variation and inseparable spatiotemporal variation in disease risk respectively based on Integrated Nested Laplace Approximation (INLA) to investigate the spatiotemporal characteristics of human rabies across 31 provinces in China from 2004 to 2020. It also investigated the influence of natural and socio-environmental factors on the incidence of the disease. Within the study period, a total of 26,807 cases of human rabies were reported, with the highest risk of incidence occurring in 2007, followed by a steady annual decline to the lowest risk in 2020. Guangxi Province exhibited the highest risk, while Jilin Province had the lowest, with the southern, central, and eastern regions reporting higher risks than the northern and western areas. By 2020, most provinces such as Guangxi and Guizhou had significantly reduced their relative risk (RR) of human rabies from historical highs. However, some provinces like Hunan, Henan, and Jiangsu experienced an increase in *RR* compared to previous years. As the annual average temperature increases, the risk of human rabies incidence in China correspondingly rises. Conversely, with increases in the annual average daily sunshine duration, per capita disposable income of urban residents, and local government healthcare expenditures, the risk of human rabies incidence declines. We conclude that the risk of human rabies in China initially increased and then decreased annually from 2004 to 2020. Future efforts should continuously increase financial investments in rabies prevention and control, focusing particularly on Hunan, Henan, Jiangsu, and provinces characterized by higher temperatures, shorter sunshine durations, and lower economic levels.

## Author summary

Rabies is a Neglected Tropical Disease (NTD), predominantly prevalent in Africa and Asia, with a nearly 100% fatality rate upon onset. China is striving to achieve the WHO’s global goal of zero deaths from dog-mediated human rabies by 2030. Therefore, exploring the spatial and temporal evolution of human rabies in China and the possible influencing factors can provide a scientific basis for the development of more effective human rabies prevention and control programs in China. The results of the study showed that from 2004 to 2020, the overall relative risk of human rabies incidence in China showed an increasing and then decreasing trend. The relative risk of most provinces, such as Guangxi and Guizhou, declined significantly, but there were still some provinces, such as Hunan and Henan, where the relative risk showed a rising trend year by year. The results of the analysis of influencing factors indicate that lower per capita disposable income of urban residents, local government healthcare expenditures and sunshine hours, higher temperatures are associated with a higher risk of human rabies.

## 1. Introduction

Rabies, commonly manifested as acute encephalitis, is a zoonotic disease caused by the rabies virus (RABV) genotype 1 and is also categorized as a neglected tropical disease (NTD) [1]. The disease is predominantly transmitted through bites or scratches from animals infected with rabies virus [2], with 99% of human cases resulting from dog bites. [3]. The incubation period typically ranges from one to three months, and once symptoms appear, the fatality rate is nearly 100% [4]. While rabies is present on all continents except Antarctica, more than 95% of deaths occur in Asia and Africa [3]. In China, over 95% of human rabies cases are due to dog bites [5].

Recognizing the ongoing prevalence of rabies in many countries, the World Health Organization (WHO), the World Organisation for Animal Health (WOAH), the Food and Agriculture Organization of the United Nations (FAO), and the Global Alliance for Rabies Control (GARC) formed the United Against Rabies (UAR) collaboration in 2015. This alliance is dedicated to eradicating human rabies transmitted by dogs and aims to achieve zero human rabies deaths by 2030 [6,7]. Historically, China’s incidence of human rabies during the early 21st century was second globally only to India, with three epidemic peaks recorded in the mid-1950s, mid-1980s, and again at the turn of the century [8]. Despite a decline in reported cases following the third peak, achieved through measures such as training health workers in post-exposure prophylaxis (PEP) and increasing access to PEP in rural areas, rabies remains a significant public health issue in China [9].

McIntyre et al. has shown that zoonotic pathogens are more sensitive to climate change than non-zoonotic pathogens [10]. Additionally, changes in land use can alter the transmission risk of infectious diseases by modifying the living conditions for hosts and vectors, thereby increasing the opportunities for contact between wildlife and humans [11]. Previous studies have indicated that higher temperatures and rainfall are associated with increased incidence rates of human rabies [12–15], whereas higher humidity levels tend to correlate with lower incidence rates [12]. This suggests that environmental factors significantly influence rabies outcomes. Infectious diseases are intricately linked to social determinants; factors such as inadequate sanitation, restricted access to healthcare, and insufficient health education significantly contribute to the prevalence of infectious diseases. Additionally, sociodemographic factors influence exposure to infectious diseases; for instance, human rabies is predominantly observed in children aged 5–14 years [3]. In the countries affected by NTDs, more than 70% are low or lower-middle-income nations [16]. NTDs, along with HIV/AIDS, tuberculosis, and malaria, are collectively referred to as infectious diseases of poverty (IDoP) [17]. Previous studies have indicated that rabies is a disease closely associated with social determinants. A study in Vietnam indicated that higher human population density and illiteracy rates are associated with an increased risk of human rabies [12]. Similarly, research from Yunnan Province in China found a positive correlation between human rabies incidence and population density, and a negative correlation with economic levels [15]. Additionally, a study from Curitiba in Brazil demonstrated that higher economic levels are associated with lower rates of dog bites [18].

In the present study, the Bayesian spatiotemporal model was developed to analyze the spatiotemporal patterns of human rabies incidence in China from 2004 to 2020. Compared to frequentist methods, Bayesian approaches utilize Bayes’ theorem to integrate prior distributions with likelihood functions, resulting in posterior distributions. This method effectively quantifies the uncertainties of estimates and offers more reliable outcomes when handling data from small samples [19,20]. The Bayesian spatiotemporal model leverages this approach to consider the relationships between spatial and temporal correlations, thereby enabling more precise identification of high-risk areas and facilitating the creation of disease risk maps [21]. It also enables the analysis of spatiotemporal trends and potential influencing factors. This provides a scientific basis for formulating effective strategies for the prevention and control of human rabies in China, aiming to achieve the goal of zero human rabies deaths by 2030. The study utilizes Integrated Nested Laplace Approximations (INLA) for the computation of the joint posterior distribution of model parameters, a method proposed by Rue et al. [22], which effectively addresses the time-consuming and slow convergence issues associated with Markov Chain Monte Carlo (MCMC) methods.

## 2. Data sources and methods

### 2.1 Variable selection

Human rabies is a disease linked with economic and social factors [12,23]. In China, human rabies predominantly affects economically disadvantaged rural areas where both cultural and economic levels are generally low, with farmers constituting the primary affected group. There is also a seasonal pattern, with higher incidence rates observed during the summer and autumn seasons [24]. Additionally, human rabies cases are more prevalent in the warm and humid southern regions of China, whereas the northeastern regions, characterized by lower population density and longer winters, exhibit lower incidence rates [25]. Consequently, this study incorporates socioeconomic indicators: per capita Gross Domestic Product (GDP) (in ten thousand yuan), per capita disposable income of urban residents (in ten thousand yuan), and per capita disposable income of rural residents (in ten thousand yuan), expenditure on healthcare by local governments (in 100 million yuan), population density (people/sq km), urbanization rate (%), density of healthcare institutions (per 10,000 people), and the percentage of the illiterate population among those aged 15 and over (%). Meteorological factors considered are: annual average temperature (°C), annual average relative humidity (%), annual sunshine duration (hours), and annual average precipitation (mm).

### 2.2 Data sources

The Internet-based Nationwide Notifiable Infectious Disease Reporting Information System was launched in 2004, hence this study spans from 2004 onwards. Data on human rabies cases from 2004 to 2020 across 31 provinces in China (excluding Taiwan, Hong Kong, and Macao Special Administrative Regions) were obtained from the Data-center of Public Health Science (https://www.phsciencedata.cn). Economic, social, and demographic data were sourced from the annual China Statistical Yearbook (https://www.stats.gov.cn/sj/ndsj/). The year-end population figures for each province were derived from the China Statistical Yearbooks spanning 2016 to 2021, with census data specifically for the years 2010 and 2020, and estimates from official population sampling surveys used for the intervening years. Provincial area data were obtained from the Ministry of Civil Affairs of the People’s Republic of China (https://www.mca.gov.cn/). Meteorological data were sourced from the China Meteorological Data Service Centre’s China’s surface climate data daily value data set (V3.0).

### 2.3 Data processing and model building

#### 2.3.1 Data processing

The per capita GDP of each province was calculated by dividing the provincial GDP by the end-of-year population count. Population density was determined by dividing the total end-of-year population by the provincial area. The density of healthcare institutions is calculated as the number of healthcare institutions at the end of the year divided by the population at year-end [26]. Meteorological data are initially averaged annually from daily records and then interpolated using the inverse distance weighting method. Subsequently, the data are amalgamated according to administrative divisions to produce province-specific datasets.

#### 2.3.2 Variable screening

Initially, we employed Spearman’s rank correlation analysis to identify variables associated with the incidence of human rabies in China, excluding those with a *P*-value > 0.05. Subsequently, a multicollinearity analysis was performed, and variables with a Variance Inflation Factor (VIF) ≥ 10 were excluded. The final variables included in the Bayesian spatiotemporal model were standardized by subtracting the mean and dividing by the standard deviation.

#### 2.3.3 Spatial autocorrelation analysis

For each year, we conducted a global spatial autocorrelation analysis of human rabies incidence rates across China to understand the spatial clustering patterns of the disease nationwide. This analysis utilized Moran’s *I* index as the statistic for assessing spatial autocorrelation, with significance determined through *Z*-value and *P*-value. Moran’s *I* value range from -1 to 1, with values > 0 indicating positive spatial autocorrelation (closer to 1 implies stronger autocorrelation), values < 0 indicating negative spatial autocorrelation (closer to -1 implies stronger negative autocorrelation), and values close to 0 or *P*-value > 0.05 indicating a random distribution of human rabies incidence [27].

#### 2.3.4 Establishment and evaluation of the Bayesian spatiotemporal models

It is generally accepted that human rabies case data follow a Poisson distribution [28]. The model was expressed as follows for

Yit∼Poisson(Eitθit)

*i* = 1,..,31 for the 31 provinces and *t* = 1,..,17 for the years considered. *Y*_*it*_ represents the number of human rabies cases in the *i*^*th*^ province in year *t*, and *E*_*it*_ denotes the expected number of cases, which is the product of the overall incidence rate and the population of the province [29,30]. *θ*_*it*_ represents the relative risk (RR) of incidence in the *i*^*th*^ province in year *t*. Bayesian spatiotemporal model with separable spatial and temporal variation and spatio-temporal interaction models with inseparable spatiotemporal variation in disease risk are constructed [31]. These interaction models enhance the simple Bayesian spatiotemporal model framework by including spatio-temporal interaction effects, thus providing the model with the flexibility to accommodate temporal and spatial variations *RR* of human rabies. This adaptability ensures that the model captures the varying trends across different provinces and times more accurately [32]. The expressions for these models are shown in Table 1.

**Table 1:** Types of Bayesian spatiotemporal models.

Model	Formula	Interacting parameters
Spatiotemporal Model	log(θit)=b0+ui+υi+γt+ϕt	-
Spatio-temporal Interaction Model		
Type I	log(θit)=b0+ui+υi+γt+ϕt+δit	*Rδ*~*Rυ*⊗*Rϕ*
Type II	log(θit)=b0+ui+υi+γt+ϕt+δit	*Rδ*~*Rυ*⊗*Rγ*
Type III	log(θit)=b0+ui+υi+γt+ϕt+δit	*Rδ*~*Ru*⊗*Rϕ*
Type IV	log(θit)=b0+ui+υi+γt+ϕt+δit	*Rδ*~*Ru*⊗*Rγ*

-: not applicable

Here, *b*_*0*_ represents the intercept, *u*_*i*_ is the spatial structured effect reflecting spatial dependency, indicating that neighboring provinces have similar incidence rates and follows an intrinsic conditional autoregressive (iCAR) process, specified as $u_{i}{|u}_{-i}∼\mathrm{Normal}\left(\frac{1}{N_{i}}\sum_{j=1}^{n}a_{ij}u_{i},\frac{\sigma_{u}^{2}}{N_{i}}\right)$; *υ*_*i*_ is the spatial unstructured effect indicating spatial heterogeneity and follows a normal distribution, specified as $\upsilon_{i}∼\mathrm{Normal}(0,\sigma_{\upsilon }^{2})$; *γ*_*t*_ is the temporal structured effect following a first-order autoregressive (AR1) process, specified as $\gamma_{t}|\gamma_{t-1},\rho ,\sigma_{\gamma }^{2}∼\mathrm{Normal}(\rho \gamma_{t-1},\sigma_{\gamma }^{2})$,where |*ρ*|<1 is the autoregressive coefficient; *ϕ*_*t*_ is the temporal unstructured effect following a normal distribution, specified as $\phi_{t}∼\mathrm{Normal}(0,\sigma_{\phi }^{2})$; *δ*_*it*_ represents the spatio-temporal interaction effect. Since the spatial and temporal effects are divided into structured and unstructured effects, the Bayesian spatio-temporal interaction model is further divided into four types [31]. Type I model is the interaction of temporal and spatial unstructured effects, so *δ*_*it*_ follows a normal distribution; Type II model is the interaction of temporal structured and spatial unstructured effects, so *δ*_*it*_ follows AR1⊗Normal; Type III model is the interaction of spatial structured and temporal unstructured effects, so *δ*_*it*_ follows iCAR⊗Normal; Type IV model is the interaction of temporal and spatial structured effects, so *δ*_*it*_ follows iCAR⊗AR1. For the Bayesian spatiotemporal model, where disease risk is considered to have separable spatial and temporal variations, the spatial effect, denoted as $RR_{\mathrm{spatial}}=\exp(u_{i}+\upsilon_{i})$, and the temporal effect, denoted as $RR_{\mathrm{temporal}}=\exp(\gamma_{t}+\phi_{t})$; in models where disease risk features inseparable spatiotemporal interaction, the spatiotemporal interaction effect is denoted as $RR_{\mathrm{spatial}‐\mathrm{temporal}}=\exp(\delta_{it})$. We assign each random effect the penalized complexity priors proposed by Simpson et al[33], with prior parameter values using the default settings in R-INLA.

In assessing the fit of spatiotemporal interaction models within the Bayesian framework, two commonly used metrics are employed: the Deviance Information Criterion (DIC), the Watanabe-Akaike Information Criterion (WAIC)[34]. Lower values of these criteria indicate a better model fit, taking into account the complexity of the model.

#### 2.3.5 Cold and hot spot analysis

Based on the results of the Bayesian spatiotemporal model, hot spot, cold spot and non-cold and non-hot regions can be delineated according to the posterior probability of the spatial effects [35]. Hot spots are defined as provinces where the posterior probability $P(\exp(u_{i}+\upsilon_{i})>1)\geq 0.8$. Cold spots are defined as provinces where the posterior probability $P(\exp(u_{i}+\upsilon_{i})>1)<0.2$. Regions that are neither hot spots nor cold spots are those where the posterior probability $0.2\leq P(\exp(u_{i}+\upsilon_{i})>1)<0.8$.

#### 2.3.6 Ecological regression

Following variable selection, covariates were incorporated into spatiotemporal and spatio-temporal interaction models. The model with the lowest DIC and WAIC was selected to explore the determinants of human rabies incidence in China. The expressions for these models are as follows:

$$\log(\theta it)=b0+\sum_{z=1}^{Z}\beta zX\mathrm{zit}+\mu i+\upsilon i+\gamma t+\phi t$$

$$\log(\theta it)=b0+\sum_{z=1}^{Z}\beta zX\mathrm{zit}+\mu i+\upsilon i+\gamma t+\phi t+\delta it$$

Here, *β*_*z*_ represents the coefficient for covariate *z*, and *X*_*zit*_ represents the value of the *z*^*th*^ influencing factor in province *i* in year *t*. The *RR* of the influencing factors is given by exp (*β*) [36].

#### 2.3.7 Statistical software

Spearman correlation analyses were conducted using the ‘psych’ package in R software version 4.3.0. Multicollinearity analysis were performed using the ‘car’ package. Calculations of the expected number of disease cases were carried out with the ‘SpatialEpi’ package[29, 37]. The Bayesian spatiotemporal models were constructed using the ‘INLA’ package (version: 23.11.26). Data visualization and global spatial autocorrelation analyses were performed using ArcGIS 10.8 software. The significance level was set at α = 0.05.

## 3. Results

### 3.1 Epidemiological characteristics of human rabies in China’s provinces

**Fig 1:**
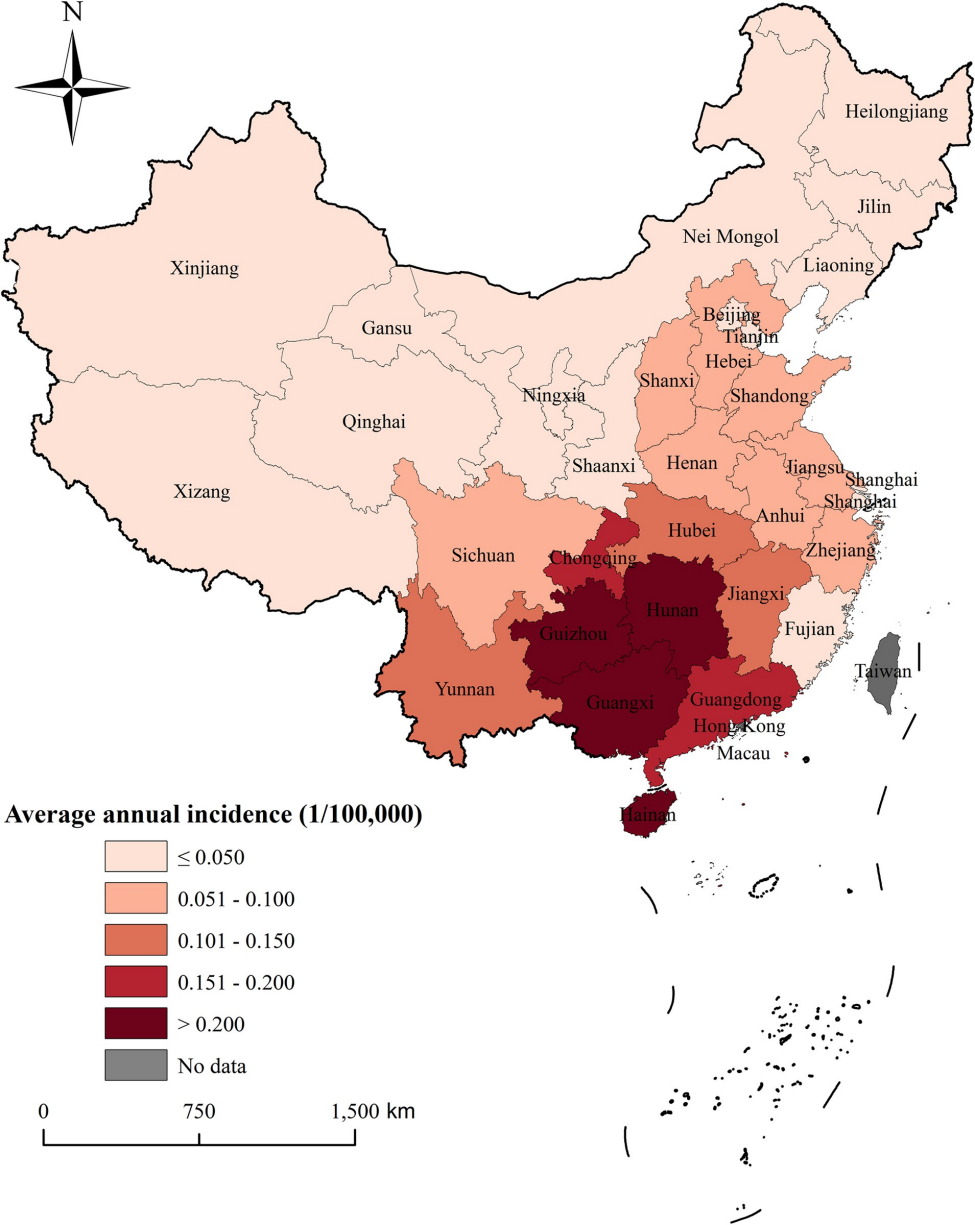
Annual average incidence rates of human rabies in provinces from 2004–2020. This map was created using ArcGIS 10.8 software. The base layer of the map is from the National Catalogue Service For Geographic Information of the Ministry of Natural Resources of the People’s Republic of China(https://www.webmap.cn/mapDataAction.do?method=forw&resType=5&storeId=2&storeName=%E5%9B%BD%E5%AE%B6%E5%9F%BA%E7%A1%80%E5%9C%B0%E7%90%86%E4%BF%A1%E6%81%AF%E4%B8%AD%E5%BF%83&fileId=BA420C422A254198BAA5ABAB9CAAFBC1).

From 2004 to 2020, China’s 31 provinces reported a total of 26,807 human rabies cases, with 26,165 fatalities. The average annual incidence rate was 0.119 per 100,000 population, and the average annual mortality rate was 0.116 per 100,000. The highest number of reported cases and deaths occurred in the years 2006–2007, with a subsequent yearly decline. The annual average incidence rates by province are shown in Fig 1, with the highest rates observed in Guangxi Province (0.52 per 100,000) and Guizhou Province (0.51 per 100,000). Guangxi, Guizhou, Hainan, and Hunan provinces had notably higher average annual incidence rates compared to other provinces. See Fig 1 for details.

### 3.2 Spatial autocorrelation analysis of human rabies

A global spatial autocorrelation analysis of human rabies incidence rates was conducted. The results indicated positive spatial autocorrelation in the incidence of human rabies in China’s 31 provinces from 2004 to 2019, manifesting as "high-high" and "low-low" clustering patterns (Moran’s *I* > 0 and *P* < 0.05). In 2020, the incidence of human rabies was found to be randomly distributed in space (Moran’s *I* = 0.102, *P* = 0.117). See Table 2.

**Table 2:** Global spatial autocorrelation analysis results of human rabies incidence rates in China’s 31 provinces from 2004–2020.

Year	Moran’s *I*	*Z*-value	*P*-value
2004	0.421	4.505	<0.001
2005	0.382	4.108	<0.001
2006	0.38	4.306	<0.001
2007	0.452	4.348	<0.001
2008	0.313	3.266	0.001
2009	0.464	4.415	<0.001
2010	0.368	3.651	<0.001
2011	0.440	4.244	<0.001
2012	0.433	4.324	<0.001
2013	0.368	3.566	<0.001
2014	0.336	3.202	0.001
2015	0.471	4.523	<0.001
2016	0.488	4.54	<0.001
2017	0.575	5.174	<0.001
2018	0.557	5.222	<0.001
2019	0.529	4.917	<0.001
2020	0.102	1.569	0.117

### 3.3 Results of the Bayesian spatiotemporal model

#### 3.3.1 Analysis of spatial effects

**Fig 2:**
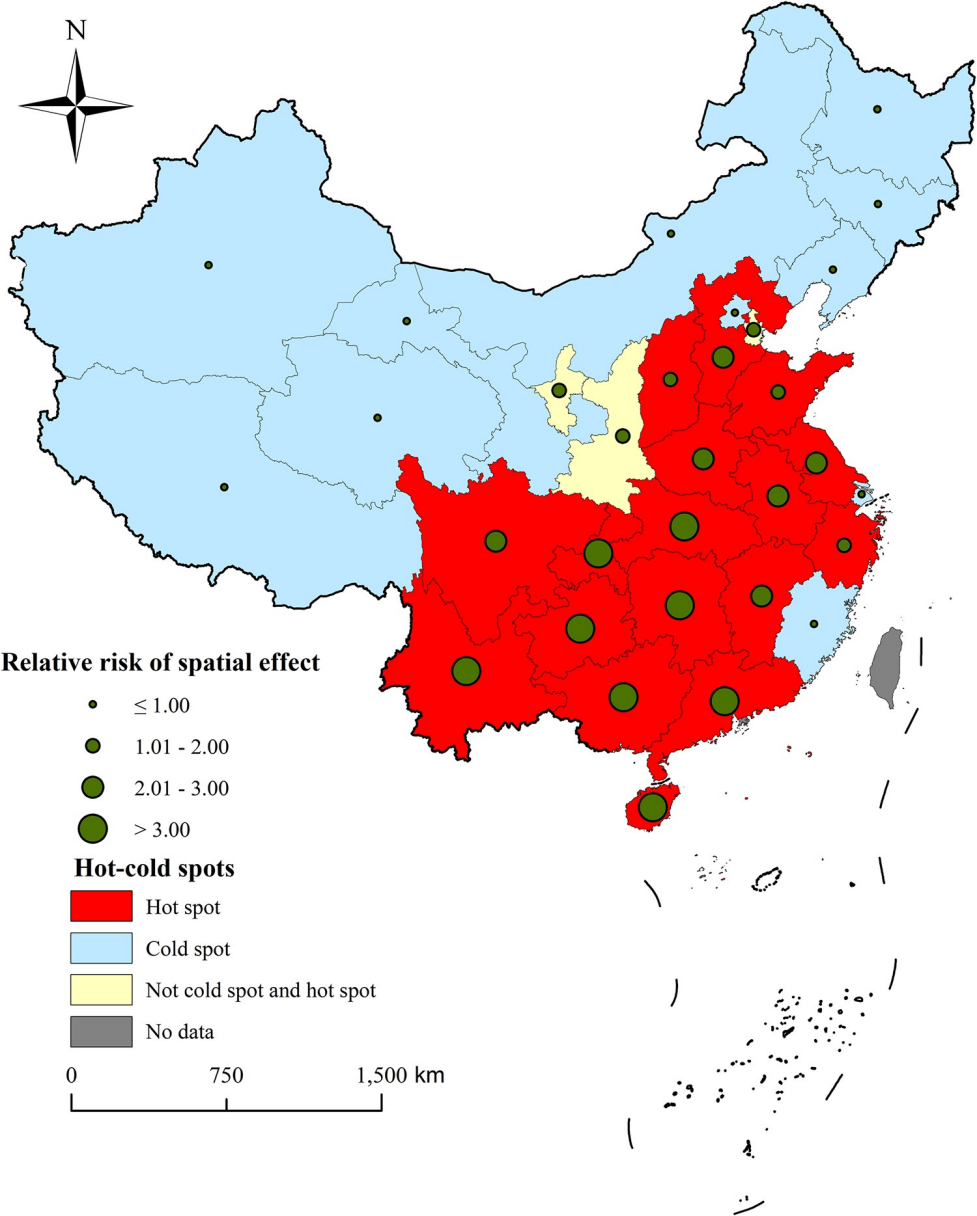
*RR* of the spatial effect and distribution of hot-cold spots. This figure illustrates the posterior means of spatial relative risks obtained from spatial random effects and the classification into hot and cold spots based on these posterior means. The size of the spheres indicates the magnitude of the spatial relative risks, with larger spheres denoting higher risks. This map was created using ArcGIS 10.8 software. The base layer of the map is from the National Catalogue Service For Geographic Information of the Ministry of Natural Resources of the People’s Republic of China(https://www.webmap.cn/mapDataAction.do?method=forw&resType=5&storeId=2&storeName=%E5%9B%BD%E5%AE%B6%E5%9F%BA%E7%A1%80%E5%9C%B0%E7%90%86%E4%BF%A1%E6%81%AF%E4%B8%AD%E5%BF%83&fileId=BA420C422A254198BAA5ABAB9CAAFBC1).

The *RR* for spatial effects was highest in Guangxi Province (13.46) and lowest in Jilin Province (0.026). The hot spot and cold spot analysis revealed 17 hot spot areas (54.84%), predominantly in the southern, central and eastern provinces, and 11 cold spot areas (35.48%), mainly in the northern and western provinces. The 3 areas (9.68%) were neither hot spots nor cold spots, namely Ningxia Province, Shanxi Province, and Tianjin. See Fig 2 for details.

#### 3.3.2 Analysis of temporal effects

**Fig 3:**
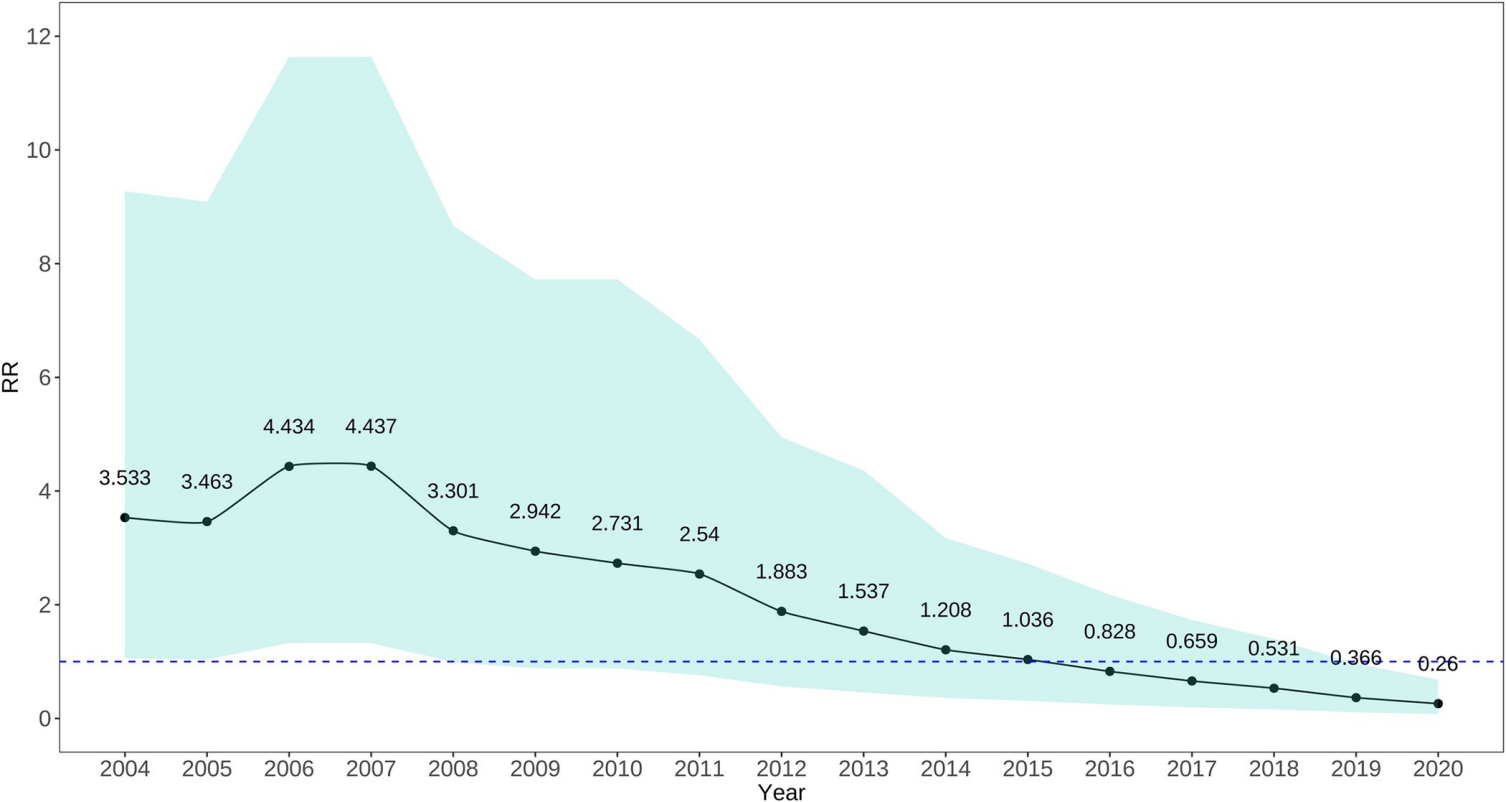
*RR* of the temporal effect for human rabies in China from 2004–2020. This figure illustrates the posterior means of the temporal relative risks for human rabies derived from temporal random effects from 2004 to 2020, presented as black lines. The 95% credible intervals (CI) are shown as blue areas.

The *RR* for temporal effects showed an initial increase followed by a decline from 2004 to 2020. The *RR* increased yearly from 2004 to 2007, reaching their peak in 2007. The period of 2006–2007 showed overall higher *RR*. Starting from 2008, the *RR* began to decrease annually, reaching their lowest level in 2020. See Fig 3 for details.

### 3.4 Results of the Bayesian spatio-temporal interaction model

The results show that the fitting effect of spatio-temporal interaction model II and IV is better than the other two, and the WAIC and DIC of model II are the lowest, so model II is finally selected for analysis. See Table 3 for the evaluation indexes of fitting performance of spatio-temporal interaction model.

**Table 3:** Evaluation of the fitting performance of spatio-temporal interaction models.

Model	Interaction	WAIC	DIC
I	*Rδ*~*Rυ*⊗*Rϕ*	3001.07	3022.13
II	*Rδ*~*Rυ*⊗*Rγ*	2811.22	2869.78
III	*Rδ*~*Ru*⊗*Rϕ*	2973.54	3006.33
IV	*Rδ*~*Ru*⊗*Rγ*	2818.56	2875.52

**Fig 4:**
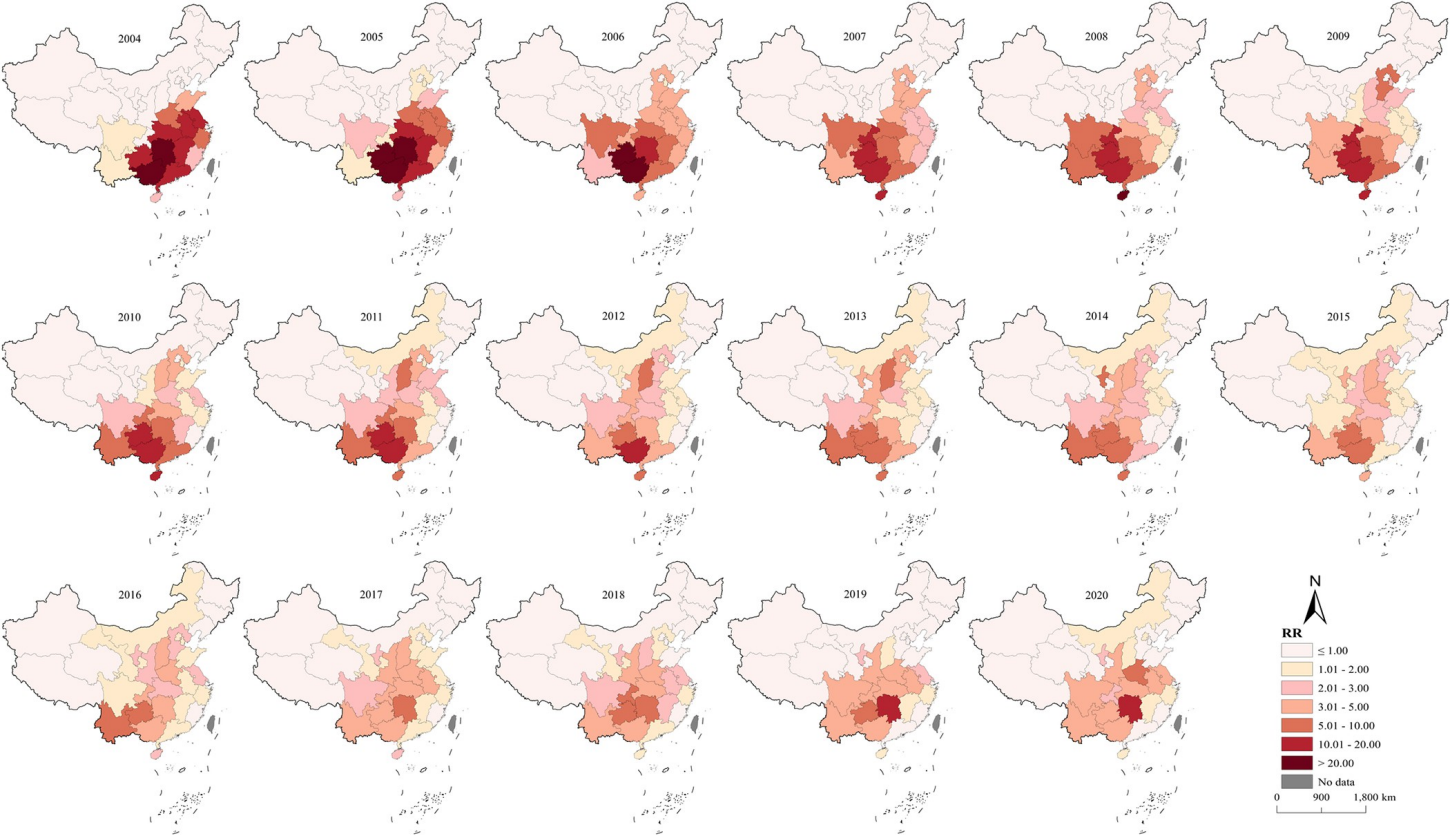
*RR* of the spatio-temporal interaction effect for Human Rabies in China from 2004–2020. This figure illustrates the posterior means of the spatio-temporal *RR* for human rabies, derived from the spatio-temporal interaction effects covering the period from 2004 to 2020. This map was created using ArcGIS 10.8 software. The base layer of the map is from the National Catalogue Service For Geographic Information of the Ministry of Natural Resources of the People’s Republic of China(https://www.webmap.cn/mapDataAction.do?method=forw&resType=5&storeId=2&storeName=%E5%9B%BD%E5%AE%B6%E5%9F%BA%E7%A1%80%E5%9C%B0%E7%90%86%E4%BF%A1%E6%81%AF%E4%B8%AD%E5%BF%83&fileId=BA420C422A254198BAA5ABAB9CAAFBC1).

The spatio-temporal interaction effects of *RR* are shown in Fig 4. The results show that from 2004 to 2007, the high-risk areas were concentrated in the southern, central, and eastern provinces, with notably higher *RR* in Guangxi, Guizhou, and Hunan provinces compared to other provinces. Starting from 2009, the epidemic area expanded gradually, but the overall *RR* showed a year-by-year declining trend. In 2020, only 2 provinces had an *RR* greater than 5, an 80% decrease from 2005 (10 provinces). These two provinces were Hunan (16.65) and Henan (5.31). The *RR* of human rabies in Hunan Province has increased yearly since 2015 and has become the province with the highest *RR* in China from 2017. The *RR* in Henan Province has shown a fluctuating upward trend since 2011. Besides, the *RR* in Sichuan and Jiangsu provinces has increased year by year since 2017 and 2018, respectively. The *RR* of Guangxi Province decreased by 90.73% from its historical high, while that of Guizhou Province decreased by 90.41%. Additionally, significant declines were also observed in Chongqing, Hainan, and Guangdong provinces.

### 3.5 Ecological regression

The collinearity analysis revealed that the per capita GDP, per capita disposable income of rural residents and annual average humidity exhibited VIF greater than 10, indicating significant multicollinearity. Consequently, these variables were excluded from further analysis. The final variables incorporated in model for analysis included: per capita disposable income of urban residents, expenditure on healthcare by local governments, percentage of illiterate population to total aged 15 and over, density of healthcare institutions, urbanization rate, population density, annual sunshine duration, annual average temperature, and annual average precipitation.

The results indicate that, after incorporating covariates, the Type II spatio-temporal interaction model exhibited the lowest DIC and WAIC, suggesting optimal model fit. Consequently, this model was selected to investigate the influencing factors of human rabies incidence in China. See Table 4 for details.

**Table 4:** Ecological regression model fitting results.

Model	Interaction	WAIC	DIC
Spatiotemporal Model	-	8339.73	5539.74
Spatio-temporal Interaction Model			
I	*Rδ*~*Rυ*⊗*Rϕ*	2984.44	3010.46
II	*Rδ*~*Rυ*⊗*Rγ*	2811.24	2868.43
III	*Rδ*~*Ru*⊗*Rϕ*	2981.00	3009.24
IV	*Rδ*~*Ru*⊗*Rγ*	2817.51	2872.63

-: not applicable

The results of the influencing factor analysis indicated a positive correlation between the risk of human rabies and annual average temperature (*RR* = 3.381, 95% *CI*: 2.275–4.838), implying a higher risk with increasing temperature. A negative correlation was observed with per capita disposable income of urban residents (*RR* = 0.625, 95% *CI*: 0.410–0.917), expenditure on healthcare by local governments (*RR* = 0.763, 95% *CI*: 0.572–0.996) and annual sunshine duration (*RR* = 0.668, 95% *CI*: 0.533–0.826), indicating that higher income levels, greater local governments healthcare spending, and longer sunshine duration are associated with lower risks. Other variables did not show a statistically significant correlation with the risk of human rabies (95% *CI* included 1). See Table 5 for details.

**Table 5:** *RR* and 95% Credible Intervals of influencing factors in Type II spatio-temporal interaction model.

Variable Type	Influencing Factor	*RR*	95% *CI*
-	Intercept	0.172	0.083–0.297
Socioeconomic Factor	Per Capita Disposable Income of Urban Residents	0.625	0.410–0.917
	Expenditure on Healthcare by Local Governments	0.763	0.572–0.996
	Urbanization Rate	0.977	0.786–1.200
	Population Density	1.077	0.680–1.633
	Density of Healthcare Institutions	1.24	0.993–1.537
	Percentage of Illiterate Population to Total Aged 15 and Over	0.917	0.728–1.139
Environmental Factor	Annual Sunshine Duration	0.668	0.533–0.826
	Annual Average Precipitation	0.969	0.833–1.121
	Annual Average Temperature	3.381	2.275–4.838

-: not applicable

## 4. Discussion

This study investigated the spatiotemporal distribution and potential influencing factors of human rabies in China from 2004 to 2020. The results indicate that from 2004 to 2019, cases of human rabies in China exhibited spatial clustering, with higher risks observed in the southern, central, and eastern provinces compared to the northern and western provinces. In 2020, the spatial distribution of human rabies cases in China appeared random, which may be attributed to reduced human mobility due to the COVID-19 pandemic, subsequently diminishing exposure risks. From 2004 to 2020, the *RR* of human rabies in China initially increased, followed by a decline, with most provinces reaching a lower level of *RR* by 2020. Notably, the many provinces such as Guizhou, Guangxi, and Hainan experienced significant declines from their peak years. China’s human rabies control measures have been effective.

In 2020, the provinces with a *RR* greater than 5 included Hunan and Henan provinces. Hunan has been the province with the highest *RR* since 2017, showing a year-over-year increase. Additionally, the *RR* in Henan Province has exhibited fluctuating upward trends since 2011. Sichuan and Jiangsu provinces have seen a rebound in *RR* in recent years. Lower coverage of standardized post-exposure prophylaxis and canine immunization, and lack of public awareness of rabies are potential reasons for the continued high risk of rabies in humans in these provinces [38–40].

The results regarding influencing factors show that China’s *RR* of human rabies is positively correlated with annual average temperature, consistent with previous studies [14,41] and aligning with the higher incidence of rabies during the summer and autumn seasons in China [42,43]. In warmer climates, people tend to wear lighter clothing and increase outdoor activities. Additionally, dogs are more irritable [13] and have a wider range of activities in hot weather [14], increasing the risk of bites. The study also found a negative correlation between the *RR* of human rabies and annual sunshine duration, which aligns with the higher average annual sunshine duration in northern provinces compared to the southern provinces [44], where rabies risk is higher. This association between lower risk and longer sunshine duration may reflect changes in human and vector behavior rather than the direct effects of UV light on the rabies virus. Although the rabies virus is sensitive to UV light [45], transmission primarily occurs through direct contact between humans and vectors. Sunshine duration may influence the risk of human rabies by altering the behaviors of humans and vectors, thereby impacting the likelihood of exposure.

There is a negative correlation between the *RR* of human rabies and the per capita disposable income of urban residents in China, consistent with previous findings [13], Human rabies is closely associated with economic levels, predominantly affecting impoverished populations [3]. As economic standards improve, there is typically an enhanced focus on health and an increased awareness of disease prevention, which may lead to reduced exposure risks to human rabies. Additionally, in this study, local government healthcare expenditures were found to be negatively correlated with the *RR* of human rabies in China. With the economic growth, there has been a consistent increase in government spending on healthcare, which has supported various initiatives. These include health education campaigns targeting rabies [46], inclusion of outpatient costs for dog bite victims under medical insurance [43], and provision of free rabies vaccinations for dogs and cats [47]. Additionally, human rabies vaccines, human rabies immunoglobulin, and anti-rabies serum have been included in China’s national medical insurance drug list [48], effectively promoting rabies control. A study showed that over the past 20 years, post-exposure vaccination rates and antibody injection rates have significantly increased in China, along with rising canine immunization coverage [49]. However, as of 2020, the average canine immunization coverage rate at Chinese monitoring sites was only 30% [50], while the WHO considers a coverage rate of 70% sufficient to eliminate human rabies [51]. Vaccinating dogs is considered the most cost-effective method for eliminating human rabies [52], and this method has contributed significantly to the effective control of human rabies in Guangxi Province [53].

In China, human rabies predominantly occurs in rural areas [50], but this study found no significant correlation between urbanization rate and *RR* of human rabies, aligning with the findings of Hangyu Li et al. [41]. Some provinces with lower urbanization rates, like Xizang and Xinjiang, are located in high-altitude areas characterized by low temperatures and longer sunshine duration, reducing dog activity and human exposure risk. Studies on the influencing factors of human rabies in Yunnan Province [15] and Vietnam [12] by Jing Yu and Dung Phung, respectively, identified human population density and annual average precipitation as risk factors, and annual average relative humidity as a protective factor. However, in this study, these factors did not show a significant correlation with the *RR* of human rabies, possibly because areas with high population density, such as Beijing, Shanghai, and Tianjin, are not hotspots for human rabies in China. Additionally, the spatial scale of this study was at the provincial level, and correlations may exist at smaller spatial scales.

This study has limitations. The analysis was conducted at the provincial-level spatial scale and annual temporal scale, which may introduce biases in identifying influencing factors; moreover, how each influencing factor affects the risk of human rabies requires deeper investigation. The human rabies case data were sourced from the Data-center of Public Health Science and lacked detailed individual case investigations, which could lead to biases and underreporting issues. The absence of comprehensive monitoring of canine immunization coverage and dog populations in China restricted the inclusion of these variables, limiting further exploration of potential factors influencing the incidence of human rabies. These limitations highlight areas for future research. Provinces in China should systematically monitor canine coverage and dog populations, and manage this data through a dedicated database, to better assess risks and devise more effective prevention strategies.

In summary, this study employs Bayesian spatiotemporal models to analyze the epidemiological characteristics of rabies in China from 2004 to 2020, exploring potential influencing factors. The results reveal spatial clustering of human rabies cases, with higher risks in the southern, central, and eastern provinces than in the northern and western provinces, and initially increased and then decreased in *RR* over the years. The annual average temperature was identified as a risk factor for human rabies in China, while per capita disposable income of urban residents, expenditure on healthcare by local governments and annual sunshine duration were protective factors.

Moving forward, efforts should concentrate on human rabies prevention and control in Hunan, Henan, Jiangsu provinces and other areas with high temperatures, low sunshine hours and low economic levels. It is imperative to increase financial investment in rabies control, enhance standardized treatment rates for exposed populations, strengthen surveillance, and improve canine immunization coverage to effectively manage and mitigate the risk of rabies in China.

## 5. Conclusion

This study reveals the spatial and temporal dynamics of human rabies in China from 2004 to 2020, while examining its association with socio-economic and environmental factors. Over the observed period, the risk of human rabies in China initially increased and then showed a consistent decline. Regions with higher risk were mainly located in the southern, central and eastern provinces. While most provinces in China have achieved commendable control over human rabies, there are still provinces such as Hunan and Henan where rabies cases are on the rise, highlighting them as critical areas for enhanced prevention and control measures. The analysis identifies annual average temperature as a risk factor, annual sunshine duration, per capita disposable income of urban residents and local government expenditure on healthcare as protective factors for human rabies in China. These findings suggest that increasing financial commitments to rabies prevention and focusing on provinces with high temperatures, short sunshine durations, and lower economic levels will be pivotal for future control strategies in China.

## Disclaimer

The authors alone are responsible for the views expressed in this article and they do not necessarily represent the views, decisions, or policies of the institutions with which they are affiliated.

## Supporting information

S1 Fig Correlation analysis. (TIF)

S1 Table Multicollinearity analysis. (DOCX)
