# Supplementary figures and images for: Spatiotemporal Distribution of Human Rabies and Identification of Predominant Risk Factors in China from 2004 to 2020
Source: PLoS Negl Trop Dis. 2024 Oct 31;18(10):e0012557. doi: 10.1371/journal.pntd.0012557 (PMC11527303; doi:10.1371/journal.pntd.0012557)

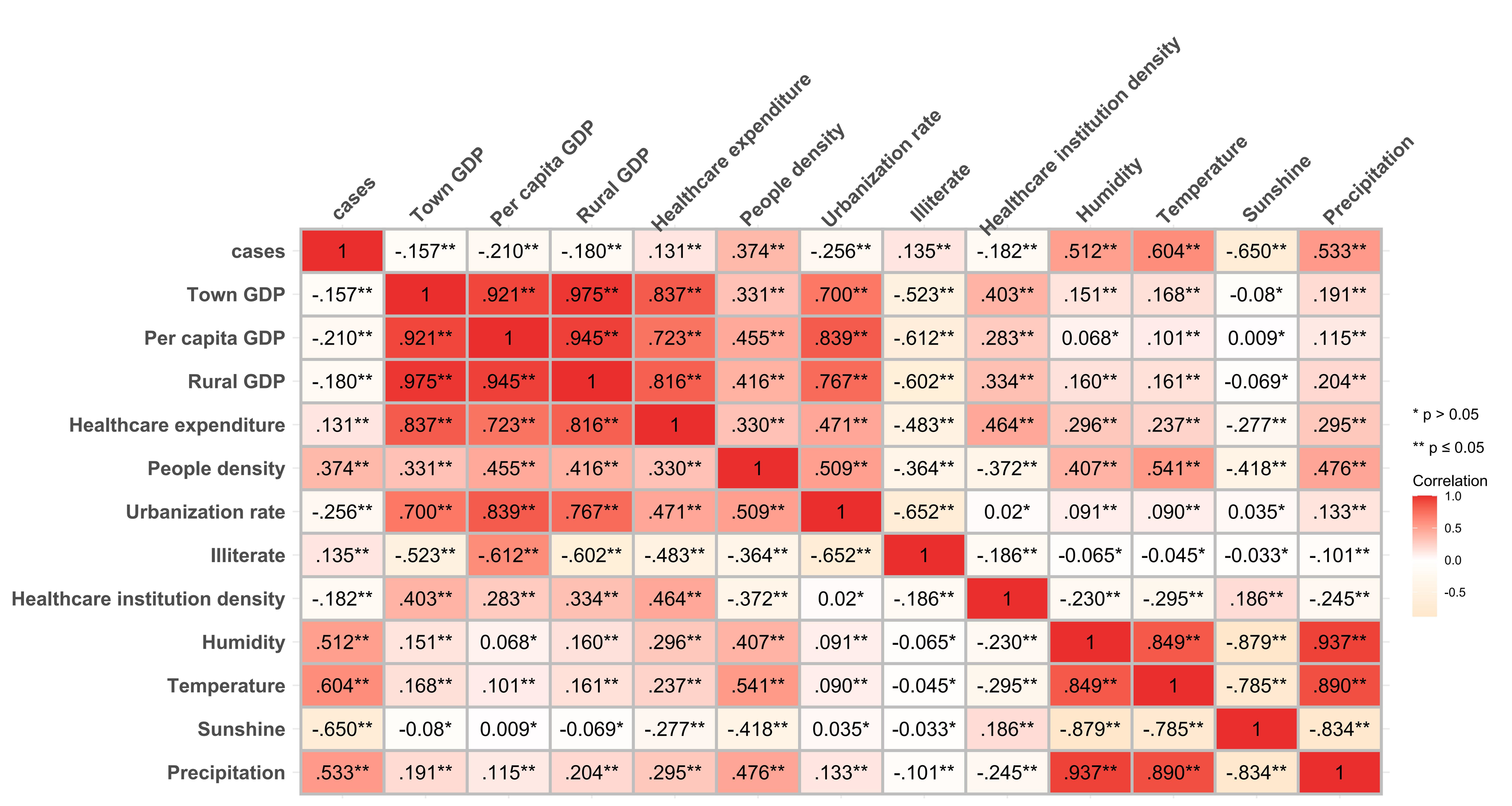

Supplement: S1 Fig — (TIF) [file pntd.0012557.s001.tif]
