# Supplementary material for: Spatiotemporal Distribution of Human Rabies and Identification of Predominant Risk Factors in China from 2004 to 2020
Source: PLoS Negl Trop Dis. 2024 Oct 31;18(10):e0012557. doi: 10.1371/journal.pntd.0012557 (PMC11527303; doi:10.1371/journal.pntd.0012557)
Supplement: S1 Table — (DOCX) [file pntd.0012557.s002.docx]

**Supporting information**

**S1 Table.** Multicollinearity analysis. We iteratively removed one variable with a variance inflation factor (VIF) greater than or equal to 10 at a time, continuing this process until all remaining variables had a VIF of less than 10.

| Influencing Factor | VIF | | | |  |
| --- | --- | --- | --- | --- | --- |
|  | Step 1 | Step 2 | Step 3 | Step 4 |  |
| Per Capita Disposable Income of Urban Residents | 13.464 | 11.180 | 4.432 | 4.422 |  |
|  |  |  |  |  |  |
| Per Capita Disposable Income of Rural Residents | **28.454** | / | / | / |  |
|  |  |  |  |  |  |
| Per Capita Gross Domestic Product | 27.941 | **12.446** | / | / |  |
|  |  |  |  |  |  |
| People Density | 2.245 | 2.222 | 2.156 | 2.155 |  |
|  |  |  |  |  |  |
| Expenditure on Healthcare by Local Governments | 2.621 | 2.610 | 2.607 | 2.501 |  |
|  |  |  |  |  |  |
| Urbanization Rate | 5.025 | 5.020 | 4.075 | 4.030 |  |
|  |  |  |  |  |  |
| Density of Healthcare Institutions | 2.050 | 2.013 | 1.977 | 1.749 |  |
|  |  |  |  |  |  |
| Percentage of Illiterate Population to Total Aged 15 and Over | 1.953 | 1.951 | 1.948 | 1.795 |  |
|  |  |  |  |  |  |
| Annual Average Humidity | 11.873 | 11.520 | **11.502** | / |  |
|  |  |  |  |  |  |
| Annual Average Temperature | 4.723 | 4.569 | 4.550 | 4.540 |  |
|  |  |  |  |  |  |
| Annual Sunshine Duration | 5.143 | 4.997 | 4.996 | 2.914 |  |
|  |  |  |  |  |  |
| Annual Average Precipitation | 7.683 | 7.659 | 7.597 | 4.468 |  |
|  |  |  |  |  |  |
